# Navigating rural challenges and promoting posttraumatic growth via expressive writing: A qualitative study among rural breast cancer survivors

**DOI:** 10.21203/rs.3.rs-9089781/v1

**Published:** 2026-04-22

**Authors:** Yusi Aveva Xu, Eunju Choi, Celia C. Y. Wong-Meli, Maria Borjas, Fei Fei, Sarah Mann, Wenyue Jin, Lenna Dawkins-Moultin, Di Lun, Qian Lu

**Affiliations:** The University of Texas MD Anderson Cancer Center

**Keywords:** expressive writing, rural breast cancer survivors, qualitative study, psychosocial intervention, cancer disparities

## Abstract

**Purpose:**

Breast cancer is the second most prevalent cancer among women in the United States. Rural breast cancer survivors (RBCS) face unique challenges because geographical isolation limits access to facilities, support groups, and mental health services. Expressive writing (EW), a therapeutic intervention navigates individuals through traumatic experiences, has demonstrated benefits for RBCS. However, first-person accounts of how RBCS experience EW remain limited. This study addresses this gap.

**Methods:**

Virtual semi-structured in-depth interviews were conducted with participants (N = 14) who previously completed a virtual EW randomized controlled trial. Interviews were audio-recorded, transcribed verbatim, and analyzed using thematic analysis.

**Results:**

Interviewees’ mean age was 54.71 years. Six themes emerged: rural-specific challenges (e.g., travel burden, infrastructure limit, gossip in small towns); emotion regulation and adaptive coping; personal strength through meaning-making; improved relationships; renewed purpose; and appreciation for life. These themes reflect participants’ narratives of how EW fit into their cancer journeys.

**Conclusions:**

Participants reported varied but positive experiences with EW. The intervention appeared to address key psychosocial needs of RBCS, helping to mitigate rural-specific disparities in supportive care and fostering elements of posttraumatic growth. Centering survivors’ own narratives adds a nuanced understanding of what it feels like to engage in EW and how the process supports adaptation in survivorship. EW may address unmet cognitive and emotional needs among RBCS, and may serve as an economical, accessible, and scalable survivorship support strategy. Future studies may triangulate the writings, self-accounts of experiences and intervention outcomes from EW interventions to contextualize the findings.

## Background

Breast cancer is the second most common cancer among women in the United States, with 310,720 new cases estimated in 2024 alone [1] and remains the second-leading cause of cancer-related mortality in women [2]. Breast cancer survivors (BCS) often face substantial physical, emotional, and cognitive challenges [3]. Rurality poses additional structural, socioeconomic, environmental, and access-to-care barriers [4] for BCS, including limited access to health care facilities, extended travel distances for care, and a scarcity of mental health resources [4, 5]. These obstacles contribute to disparities in survivorship and may exacerbate feelings of isolation, psychological distress, and unmet emotional needs among rural BCS(RBCS). Studies showed that RBCS reported higher levels of anxiety and depression than their urban counterparts [6] underscoring the need for tailored interventions to bridge these gaps in survivorship care.

Expressive writing (EW) has emerged as a psychosocial intervention designed to help individuals process trauma. Pioneered by Pennebaker and Beall [7], EW involves writing about one’s deepest thoughts and feelings surrounding traumatic life events. Studies have demonstrated EW’s efficacy in improving emotional well-being, reducing psychological distress, and enhancing quality of life among cancer survivors, particularly among those with low emotional support (e.g., rural survivors) [reviews see 8-10]. Despite these documented benefits, few studies have focused on the use of EW among rural cancer survivors [11], particularly in virtual formats that could address barriers to in-person care.

The COVID-19 pandemic created an unprecedented disruption in health care services, particularly affecting rural communities which were challenged by limited health care infrastructure [11]. This disruption heightened the demand for accessible, remote interventions. Virtual EW can alleviate travel burdens, offer scheduling flexibility, and allow participants to privately explore their emotions from home, potentially filling care gaps during periods of restricted supportive care access [13].

Prior EW studies have analyzed participants’ written essays, *none* have examined participants’ own accounts of their experiences with the intervention- particularly among rural survivors- highlighting this study’s novelty and significance. To capture insights that written texts alone cannot reveal, we conducted semi-structured interviews focused on participants’ reflections, motivations, and feelings of the writing process. Interviews offer rich contextual information about emotional states, intentions, and subjective evaluations of the intervention. Therefore, participants were interviewed after completing the intervention to elicit deeper understanding of their experiences and the personal growth attributed to EW. This study aimed to comprehensively explore how EW might facilitate psychosocial well-being and positive adaptation among RBCS, while accounting for the unique adversities associated with rural survivorship.

## Methods

### Participants

This study was approved by the institutional review board. The parent intervention was a randomized controlled trial of an online EW intervention conducted between October 2021 and April 2022. Eligible individuals were aged 18 years or older, diagnosed with any stage (0-IV) of breast cancer within the past 3 years, proficient in English, had access to internet, and resided in an area with a zip code designated as “rural” by the Federal Office of Rural Health Policy [Health Resources & Services Administration]. The parent study was registered with ClinicalTrials.gov (NCT04776941), and its detailed procedures, feasibility, and preliminary outcomes were previously reported.[16]

The research team invited participants who completed the EW intervention to this poststudy interview via email, text or calls. Informed consent was obtained from all participants.

### EW intervention

Participants in the parent study were recruited from a list of patients with breast cancer at a cancer hospital and were randomly assigned to either the EW arm (wrote about their deepest thoughts and feelings about their cancer experiences) or the control arm (wrote about neutral topics). The intervention spanned 6 weeks, with 3 writing tasks (write continuously for at least 15 min) scheduled 2 weeks apart. Appendix A documents the respective writing topics. They could either type or write on paper. Follow-up questionnaires were sent at 1, 3, and 6 months after the last writing task.

### Interview procedure and thematic analysis

In total, 14 post-study interviews were conducted in November and December 2023. Thirteen interviews were conducted via Zoom, and 1 took place in person. Each interview took 45 to 60 minutes. Participants gave verbal consent to audio recording and received gift cards as compensation. The interviews followed a semi-structured interview guide (Appendix B).

Audio recordings were transcribed, and the research team conducted a thematic analysis using an iterative process [17]. After repeatedly reading the transcripts, the first two authors inductively developed an initial codebook. Each transcript was independently coded by team members. When content not covered by the existing codes emerged, findings were discussed and the codebook was refined accordingly. Once all coauthors reached consensus, the first two authors inductively categorized the codes and summarized the themes. The themes were mapped onto the post-traumatic growth (PTG) framework as relevant, with additional themes outside of PTG reported. PTG describes the positive psychological changes individuals may experience following a highly traumatic event [14], and is well documented among cancer survivors [15]. IT includes 5 domains: increased personal strength; enhanced interpersonal relationships; recognition of new possibilities in life; heightened appreciation for life; and spiritual growth [14].

## Results

### Participant characteristics

The mean age of the interviewees was 54.71 years. Most interviewees self-identified as White (93%), whereas 1 participant (7%) identified as African American/Black. Three participants (21%) identified as Hispanic. Five participants (36%) had stage I cancer, 5 participants (36%) had stage II cancer, and 4 participants (28%) had stage III cancer. Table 1 documents additional demographic information.

### Emerging themes

Thematic analysis revealed interrelated themes, highlighting the multifaceted benefits of EW for RBCS. They were rural-specific challenges, emotion regulation and adaptive coping, and 4 of the 5 domains of the PTG framework (personal strength through meaning-making, improved relationships, renewed purpose, appreciation for life). Table 2 documents themes, the codebook and representative quotes.

### Rural-specific challenges

Participants reported challenges unique to rural living that they experienced during and after cancer treatment, including limited access to treatment facilities, slow internet connectivity, and significant travel burdens. One participant mentioned, “I live in a remote area, so we have slow internet connections [to receive virtual service]…” (ID: 123, 62 years, stage III). Another emphasized the travel burdens: “[The treatment center] It’s 3 1/2 to 4 hours from my home, and that was a weekly trip.” (ID: 196, 65 years, stage II). These accounts underscored how geographic isolation and infrastructure limitations significantly impacted participants’ cancer care experience, and therefore, their critical need for virtual care.

Rural residence also intensified privacy concerns regarding sharing their cancer experiences, and participants reported EW addressed privacy concerns: “…we live in very, very small towns, like a town of 235 people, and *everybody knows me*. [People of small towns] They're terrible gossips… and I just didn't want to be subjected to that. The study helped.” (ID: 664, 67 years, stage I).

### Emotion regulation and adaptive coping

EW served as a key strategy for emotion regulation, allowing participants to release pent-up emotions and engage in adaptive coping. Participants described the intervention as therapeutic: “It helped me release all the frustrations I had that I couldn't openly talk to anyone about.” (ID: 309, 32 years, stage III). Another participant shared, “If I can't talk to anybody, I write it down. So that's how I let it go. I use it to vent, so it's perfect. It works.” (ID: 129, 51 years, stage II).

Additionally, interviewees used EW to identify stressors and reflect on coping strategies as ways for self-management. Participants used EW to reassess their challenges and explore solutions: “[Being in the study] I was able to think about the situation, think about what made things more stressful. Being able to put it down on paper, you're able to look at it more and think about it more to help what you could do to get through that.” (ID: 217, 52 years, stage II). Another mentioned: “[Writing] was really important for me, with my lingering memory problems from chemotherapy.” (ID: 123, 62 years, stage III). Others used EW for introspection and to reconcile with their restraints: “[Through EW I realized] my whole life, the only downfall of things was my finances…” (ID: 317, 52 years, stage III). These reflections indicate that EW helped emotion regulation, problem-solving and adaptation.

### Personal strength through meaning-making

Interviewees reported gained cognitive clarity through the increased self-awareness fostered by EW. Participants enhanced awareness about their experiences and acknowledged their feelings: *“It really helped to let you know what you're going through… It helps to write down: …this is what I’m experiencing.”* (ID: 143, 54 years, stage I). Similarly, another used EW to process her experience holistically: *“It… all comes together to where I felt like I was summarizing what had happened to me, and I think I was understanding it better.”* (ID: 279, 63 years, stage I). Another mentioned “[Writing] made me more aware of the experiences. I didn’t think about them until [this intervention] asked”. (ID: 123, 62 years, stage III).

Furthermore, interviewees reinterpreted their trauma in a positive and resilient light through EW: “When I was diagnosed with cancer, basically, that just put a stop to everything… to look at any bad situation… It's not really that bad. And just, I just kind of turned it around.” (ID: 317, 52 years, stage III). Another participant even put a positive spin on their cancer experience and reframed their trauma into a source of resilience and hope: “Writing about the traumatic yet meaningful cancer experience helped me find hope and love.” (ID: 217, 52 years, stage II).

### Improved relationships to others

Aligning with the PTG framework, interviewees mentioned that EW enhanced social connectedness. Interviewees expressed reduced feeling of isolation through the recognition of shared experiences: “[EW] connected me too. Because I was thinking that other people are going through the same thing that I’m going through…” (ID: 233, 51 years, stage III). Another interviewee maintained that writing about challenges helped validate her feelings: “It’s not only my issues, it would be other people [‘s issues] too… who have the same feelings.” (ID: 129, 51 years, stage II). Additionally, strong family support was repeatedly mentioned, underscoring that writing helped survivors recognize and appreciate the importance of the interpersonal relationships that sustained them during difficult times (ID: 217, 52 years, stage II).

The data also revealed a subtheme of altruism. RBCS leveraged EW not only as a personal coping mechanism but also to support others facing similar challenges. One interviewee stated: “[I participated in EW] for altruistic reasons, just to do what I can to help someone else that’s going through breast cancer.” (ID: 143, 54 years, stage I). Another noted, “I had friends that were survivors that were a valuable resource… so if there was anything I could do to help somebody else, I was happy to do that.” (ID: 348, 61 years, stage I).

### Renewed purpose

Interviewees discovered renewed purpose during EW, reflecting the PTG’s element on new priorities after adversity. Many described a strong desire contributing to others: “I like the idea of being needed, have something meaningful to do—that’s different from being taken care of.” (ID: 123, 62 years, stage III). Another interviewee echoed this sentiment: “If there’s anything I can do to help someone else now, I’d do it.” (ID: 332, 46 years, stage II).

### Appreciation for life

Consistent with the PTG framework, interviewees reported that EW fostered gratitude and appreciation for life through self-reflection. Reflective practices during EW led interviewees to find gratitude: “I have an appreciation for just being alive and having a choice. Not everybody survives cancer.” (ID: 123, 62 years, stage III). Another noted: “There's a lot of things that I took for granted before cancer, such as just physically being able to move around like walking. And now that I've had cancer and gone through it, I'm a lot more appreciative of what I can do.” (ID: 233, 51 years, stage III).

### Spiritual growth

Notably, although spiritual growth is a recognized domain in the PTG framework, our data provided too few insights to form a theme. Only one interviewee mentioned a strengthened spiritual belief through writing: “I feel, on a spiritual part, closer to God.” (ID: 233, 51 years, stage III).

## Discussion

This qualitative study provides an in-depth account of how RBCS experienced a virtual EW intervention and its role in survivorship adaptation. Themes emerged were rural-specific challenges, emotion regulation and adaptive coping, personal strength through meaning-making, improved relationships to others, new directions, and appreciation for life. These themes suggest EW may function as an accessible, low-cost communication-based supportive care that well matched to rural contexts where structural barriers to supportive care and social constraints on disclosure intensifies survivorship distress. EW affects people on a cognitive, emotional, social, and biological level, making a single explanatory theory for EW unlikely.

### Rural-specific challenges: structural and social constraints

Interviewees’ accounts of long travel distance to service and time burden mirror a robust body of rural survivorship research. Primarily due to geographic isolation, RBCS may experience limited access to specialty providers, a lack of one-stop services, lower access to survivorship care services, and infrastructure and technology barriers [8, 18] and elevated need for supportive care in survivorship [19]. These reported constraints constitute *persistent inequality* in survivorship as evidence suggests RBCS experience poorer prognosis despite lower incidence [3]. Beyond structural barriers, interviewees described rural community dynamics, such as worry for gossip, which may impede help-seeking and disclosure. This aligned with literature that cultural norms such as self-stigma and self-reliance reduced psychosocial service utilization, even telehealth [20] in the tight-knit rural communities [21]. Interviewees also described the internet infrastructure challenges (e.g., slow speed), which further limit access to synchronous supportive care [8] and highlighted the feasibility of EW as an accessible form of psychosocial support. In fact, a systematic review highlighted this “digital paradox” and concluded that rural survivors benefit from digital interventions, even as a digital divide persists [22].

### Emotion regulation and adaptive coping: disclosure and self-management

Interviewees described the EW as a cathartic outlet and a safe space to release pent-up emotions that might otherwise be neglected or intentionally suppressed. Before the intervention, some RBCS reported ambivalence or unresolved concerns about openly disclose emotions. They reported cherishing the EW as a private outlet to support integration of feelings into structured narratives – a key pathway to reduce avoidance and intrusions over time [23, 24].

Interviewees considered digital EW as an opportunity to recenter themselves and reflect on self-management, including identifying stressors and strategies for adaptive coping. This aligns with systematic review that EW may activate cognitive processing and self-reflection [25], and enhance goal-relevant, self-regulatory processes [26]. On a more practical note, interviewees described using EW to identify stressors and consider ways to respond. This reflects adaptation from trauma, another frequently reported domain of PTG according to a recent review [27]. Such findings highlight EW's potential as a valuable self-management tool in rural settings, particularly when traditional in-person counseling or synchronous counseling services are scarce or inaccessible.

### Meaning-making and personal strength through narrative reconstruction

Interviewees reported significant personal strength through deliberate meaning-making: they described improved awareness, cognitive clarity and perspectives shift through narrative reconstruction. Interviewees regarded EW facilitated deeper introspection, and as they explored, articulated, and confronted traumatic experiences, they formulated coherent narratives that aid understanding of their cancer journey [28] and initiated healing, which aligns with the process to achieve PTG [29, 30]. Importantly, traumatic events alone are insufficient to cause PTG [16], and such meaning-make and narrative reconstruction characterized by “deliberate rumination” may be a strong precursor of PTG [31]. As EW facilitates cognitive restructuring [24], and the reconstruction helps survivors frame their trauma in a meaningful and resilient manner [32]. In fact, interviewees reported finding hope, love, and positivity amidst EW, and they reported developing a greater sense of personal strength and boosted confidence in their ability to overcome adversity, which fits PTG and reinforces the value of qualitative work capturing survivors’ own accounts of growth processes.

### Improved social relations: connectedness and altruism

Interviewees reported that EW helped them feel less alone, recognize shared experiences, validate feelings and strengthen relationships, echoing a relational pathway that *intrapersonal* processing support *interpersonal* relations [33]. The improved interpersonal relationships and social connectedness are key positive changes in PTG [14] and align with findings that ethnic minority BCS reported improved social relationships amid PTG [34]. Similarly, interviewees reported feeling closer to family and support systems, highlighting their perceived PTG through EW. The maintenance of such satisfying social bonds has been, in return, found to predict resilience and positive coping [17]. The altruism subtheme- interviewees framed their participation in the intervention as “helping others”-also fits the PTG framework, especially after cancer. A recent meta-synthesis focused on breast cancer PTG notes that survivors become more appreciative of family and friends and in some contexts develop increased empathy and altruistic behavior [35]—patterns closely mirrored in our findings.

### Renewed purpose and appreciation of life

Our interviewees reported engaging in the intervention may facilitate finding new purposes and directions, another PTG domain [5]. One interviewee mentioned that being able to provide for others and contributing meaningfully, rather than being taken care of, was a new purpose in life (ID: 123, 62 years, stage III). Another mentioned after the EW experience she realized that “continuing to make it better for somebody” (ID: 332, 46 years, stage II) to be worthwhile. Interviewees also consistently demonstrated enhanced gratitude and appreciation for life. Numerous studies have documented gratitude is increased by self-reflection after trauma, and enhanced appreciation for life is a domain in the PTG framework [25, 30]. According to a meta-analysis, these effects are critical, as gratitude has been linked to improved mental health outcomes, including reduced depression, increased life satisfaction, and better quality of life [37].

Notably, the PTG also includes spiritual changes; however spiritual growth was not widely evident among interviewees. Although spiritual growth was mentioned by one interviewee, it did not emerge as a pervasive pattern. This finding was consistent with a survey among survivors which found spiritual changes as the least pronounced domain [36].

### Implications for supportive care in rural survivorship

Results from our study suggest that EW may function as an economical, accessible, and scalable intrapersonal communication-based supportive care that partially mitigates rural disparities in supportive services access. Our findings extend current research among RBCS by showing the positive accounts of a virtual EW as a private space for disclosure and narrative reconstruction, especially relevant where rural community dynamics and structural constraints impede organic discussion and service seeking. At the same time, the digital divide remains a reported challenge for virtual supportive care among RBCS. Future virtual EW interventions may be mindful of rural infrastructure, for example, provide low-bandwidth or cellular-based options (e.g., offline writing with phone-based follow-up), embed it in patient portals, and in-person care when locally feasible. Finally, meta-analyses cautions against overgeneralizing EW’s efficacy across survivor populations and contexts, with small or null pooled effects, while moderators including social constraints appear promising to identify those benefit the most [9, 10, 38-40]. In this sense, rural survivors, a subgroup that may experience heightened disclosure constraints and limited access to psychosocial support, may benefit from targeted EW intervention that emphasizing PTG tailoring, as our findings indicated.

### Limitations

This study has limitations. First, the selection of interviewees may be biased toward engaged participants who enjoyed EW, limiting the generalizability of the findings; however, engaged participants are arguably in better positions to provide deep insights and share their experiences. Second, the composition of the sample may be homogeneous: female, predominantly White, and digitally-savvy. Survivors with limited digital literacy or no internet access—common challenges in rural areas [18]—may be underrepresented.

However, as most in-person services were disrupted during the pandemic, delivering the intervention digitally ensured that it was accessible to most participants. Thirdly, this analysis relies on self-reported perceived effect, and may be subjected to recall and reappraisal. Future studies may triangulate interviews with essays’ content and objective biomarkers to understand EW’s physiologic impact and to further clarify the mechanism driving PTG. Additionally, future research may consider including diverse racial, ethnic, age, or gender groups of rural cancer survivors to discover potential cultural nuances associated with rural adversities. Lastly, while structural and social constraints were salient in our findings, rurality is heterogeneous. Future work may examine specific rural markers as moderators that shape EW’s acceptability and effect.

## Conclusion

This study explored first-person accounts of how RBCS experienced a virtual EW intervention and its perceived role in survivorship adaptation during the pandemic. Interviewees described EW as a private, accessible outlet for emotion disclosure, self-reflection and narrative reconstruction, especially in rural structural and social constraints. Themes identified suggested that EW may facilitate multiple domains of PTG. Our findings extend previous literature by centering RBCSs’ own account of experiencing the intervention rather than solely rely on written essays or survey. Our study showed that EW may be particularly suited to rural survivorship contexts, where access to sage emotion disclosure is limited. Although EW may not be universally sufficient for all survivors, it appears to offer a scalable and accessible intrapersonal communication-based survivorship support tool that addresses unmet emotional and cognitive needs among RBCS.

## Supplementary Material

This is a list of supplementary files associated with this preprint. Click to download.

• SupplementalMaterial.docx

## Figures and Tables

**Table 1:** Interviewee demographic and clinical characteristics (N = 14).

Characteristic	N (%)
Age, years, mean (standard deviation)	54.17 (9.03)
Race	
African American/Black	1 (7)
White	13 (93)
Ethnicity	
Hispanic	3 (21)
Non-Hispanic	11 (79)
Cancer stage	
I	5 (36)
II	5 (36)
III	4 (29)
Highest education achieved	
High school degree	1 (7)
Some college or specialized training (associate degree)	4 (29)
College degree (bachelor’s degree)	5 (36)
Postgraduate degree	4 (29)
Occupation industry	
Service	2 (14)
Managerial/professional specialty	8 (57)
Other	4 (29)
Occupation type	
Full-time	7 (50)
Part-time	2 (14)
Homemaker	1 (7)
Retired	3 (21)
Other	1 (7)
Marital status	
Never married	2 (14)
Married	10 (71)
Divorced	2 (14)
Number of children	
0	4 (29)
1	1 (7)
2	4 (29)
3	4 (29)
4	1 (7)
Living situation	
Alone	3 (21)
Partner	5 (36)
Partner and children	5 (36)
Parents	1 (7)

**Table 2:** Inductively coded themes identified from interviews with rural breast cancer survivors.

Theme	Code label	Code Definition	Illustrative quotes
**Rural-specific challenges**	Rural challenges	Participants indicated unique rural specific challenges they face as rural cancer survivors	“I live in a remote area, so we have slow internet connections [to receive virtual service]… I did part of my treatment in Houston… I miss home when I was in Houston.” (ID: 123) “[Of treatment center] It’s 3 1/2 to 4 hours from my home, and that was a weekly trip. You know, for 12 weeks and then every two weeks or so. And then a few other times for surgeries and stuff.” (ID: 196)
**Emotion regulation and adaptive coping**	Releasing emotions	Participants described writing served as a cathartic outlet, enabling participants to release pent-up emotions	“It helped me release all that frustration I had. I think like typing it out, kind of like putting your thoughts on paper. It helped me release all the frustrations I had that I couldn't like openly talk to anyone about it.” (ID: 309) “If I can't talk to anybody, I write it out, and I write it down. So that's how I let it go, I see. I use it to vent, so it's perfect. It works. It works perfectly.” (ID: 129)
	Adaptive coping and self-management	Participants described using expressive writing to identify stressors and reflecting ways to address them (e.g., confrontation coping)	“I was able to think about the situation, think about what made things more stressful. Being able to put it down on paper, you're able to look at it more and think about what you could do to get through that… What could I have done different?’” *(ID: 217)* “I do think writing can be very therapeutic in lowering your stress…when we can write it out… it just helps us, especially when it’s negative, we can purge it.” (ID: 332) “[Of writing] It was really important for me, with my lingering memory problems from chemotherapy, to be able to integrate it into my schedule.” (ID: 123) “I guess my whole life, the only downfall of things was my finances, and I just chose not to stress on finances and do what I could do.” (ID: 317)
**Personal strength through meaning-making**	Cognitive clarity through awareness gaining	Participants gained insight into their experiences and recognized patterns or key events through writing	“[Being in the study] It made me more aware of the experiences. Of course, I had thoughts and feelings but just stopped to think about what they are.” (ID: 123) “It really helped to let you know what you're going through. Patients like me have a tendency to do this: I won’t say what’s going on, or I may, even when I go to the doctor sometimes, I may forget to tell them something. It helps to write down: well hey, this is what I’m seeing; this is what I’m experiencing”” (ID: 143) “It…all comes together to where I felt like I was summarizing what had happened to me, and I think I was understanding it better.” (ID: 279)
	Perspective changing through cognitive reframing	Writing reframed trauma as a source of resilience, strength and hope	*“Writing about the traumatic yet meaningful cancer experience helped me find hope and love.” (ID: 217)* “When I was diagnosed with cancer, basically, that just put a stop to everything. How I looked at things, how I looked at situations, how I look at people, even the things around me… to look at any bad situation… It's notreally that bad. And just, Ijust kind of turned itaround.” (ID: 317)
**Improved relation to others**	Enhancing social connectedness and interpersonal relationship	Participants reported writing and participating in the study helped them to feel connected and not alone	“I think it connected me too. Because I was thinking that other people are going through the same thing that I'm going through.… So we all, we all have times in our lives, no matter if it's cancer or not that we're at our stressful times.” (ID: 233) “It's not only my issues, it would be other people[‘s issues], too, I mean, who have the same feelings.” (ID: 129) “I think it’s good to do something like this to help others.… I had also had one or two [friends] that when I reached out to them in my—when I was first diagnosed—they were very helpful… I found that just being open… could help them as well.” (ID: 317)
	Altruism	Rural survivors leveraged their participation in the expressive writing intervention as a way to support others	“I had friends that were survivors that were a valuable resource… so if there was anything I could do to help somebody else, then I was happy to do that.” (ID: 348) “[I participated in the writing] for altruistic reasons, just to do what I can to help someone else that's going through this, you know, breast cancer.” (ID: 143) “Just to be a help to others.… If that could be a help to someone else, I would love to do it. We recently had a lady in a church diagnosed… I wrote about my experience, gave my testimony.” (ID: 196)
**Renewed purpose**	Finding renewed purposes	Participants discovered new purpose in life amid rural adversities	“I like the idea of being needed, have something meaningful to do that’s in a way different from being taken care of.” (ID: 123) “If there’s anything I can do to help someone else, I’d do it… just always continuing to make it better for somebody.” (ID: 332) “We all go through different things… sharing it, and maybe helping other people… This is what helped me.” (ID: 129) “I’ve already talked to somebody… I’ve written stuff for them about it…telling them I’m sorry, gave them instructions on going to MD Anderson from here… about diet… having a hard time eating.” (ID: 279)
**Appreciation of life**	Gratitude and appreciation	Participants developed gratitude and appreciation for life through self-reflection	“I have an appreciation for just being alive and having a choice. I know that not everybody survives cancer.” (ID: 123) “It helped me to be more thankful and more reflective.” (ID: 143) “I know that there's a lot of things that I took for granted before cancer, such as just physically being able to move around like walking and stuff like that. And now that I've had cancer and gone through it, I think I'm a lot more appreciative of what I can do.” (ID: 233)
**Spiritual changes** ^a^	Spiritual changes	Participants expressed their spiritual beliefs were strengthened	“I feel, on a spiritual part, is probably closer to God.” (ID: 233)

a Spiritual changes, a theoretical posttraumatic growth domain, was not a distinct theme that emerged from our dataset, although evidence of spiritual changes was found in 1 interview.

## References

[1] Cancer Facts and Statistics [Internet]. [cited 2025 Apr 15]. Available from: https://www.cancer.org/research/cancer-facts-statistics.html

[2] Breast Cancer Facts & Figs. 2024–2025. Am Cancer Soc.

[3] Anbari AB, Wanchai A, Graves R. Breast cancer survivorship in rural settings: a systematic review. Support Care Cancer. 2020;28(8):3517–31. doi:10.1007/s00520-020-05308-0 31970515

[4] Zahnd WE, Murphy C, Knoll M, Benavidez GA, Day KR, Ranganathan R, The Intersection of Rural Residence and Minority Race/Ethnicity in Cancer Disparities in the United States. Int J Environ Res Public Health. 2021;18(4):1384. doi:10.3390/ijerph18041384 33546168 PMC7913122

[5] Ratnapradipa KL, Ranta J, Napit K, Luma LB, Robinson T, Dinkel D, Qualitative analysis of cancer care experiences among rural cancer survivors and caregivers. J Rural Health. 2022;38(4):876–85. doi:10.1111/jrh.12665 35381622 PMC9492624

[6] Tulk J, Wurz A, Hou SHJ, Bender J, Schulte FSM, Eaton G, Rural-urban differences in distress, quality of life, and social support among Canadian young adult cancer survivors: A Young Adults with Cancer in Their Prime (YACPRIME) study. J Rural Health Off J Am Rural Health Assoc Natl Rural Health Care Assoc. 2024;40(1):121–7. doi:10.1111/jrh.12774 37355833

[7] Pennebaker JW, Beall SK. Confronting a traumatic event: Toward an understanding of inhibition and disease. J Abnorm Psychol. 1986;95(3):274–81. doi:10.1037/0021-843X.95.3.274 3745650

[8] van der Kruk SR, Butow P, Mesters I, Boyle T, Olver I, White K, Psychosocial well-being and supportive care needs of cancer patients and survivors living in rural or regional areas: a systematic review from 2010 to 2021. Support Care Cancer Off J Multinatl Assoc Support Care Cancer. 2022;30(2):1021–64. doi:10.1007/s00520-021-06440-1 PMC8364415 34392413

[9] Parv D. The effects of expressive writing intervention in cancer patients and survivors: A rapid umbrella review. Int J Mol Immuno Oncol. 2023;8:23–30. doi:10.25259/IJMIO_24_2022

[10] Abu-Odah H, Su JJ, Wang M, Sheffield D, Molassiotis A. Systematic review and meta-analysis of the effectiveness of expressive writing disclosure on cancer and palliative care patients’ health-related outcomes. Support Care Cancer. 2024;32(1):70. doi:10.1007/s00520-023-08255-8 38157056

[11] Ratcliff CG, Torres D, Tullos EA, Geng Y, Lu Q. A systematic review of behavioral interventions for rural breast cancer survivors. J Behav Med. 2021;44(4):467–83. doi:10.1007/s10865-020-00174-x 32813192

[12] Dilawari A, Rentscher K, Zhai W, Zhou X, Ahles T, Ahn J, Medical Care Disruptions During the First Six-Months of the COVID19 Pandemic: The Experience of Older Breast Cancer Survivors. Res Sq. 2021;rs.3.rs–416077. doi:10.21203/rs.3.rs-416077/v1 PMC8436022 34515905

[13] Nelson D, Cooke S, McLeod B, Nanyonjo A, Kane R, Gussy M. A Rapid Systematic Review on the Experiences of Cancer Survivors Residing in Rural Areas during the COVID-19 Pandemic. Int J Environ Res Public Health. 2022;19(24):16863. doi:10.3390/ijerph192416863 36554740 PMC9778689

[14] Shin-Cho LJ, Choi E, Dawkins-Moultin L, Wong CCY, Borjas M, Fei F, Feasibility and acceptability of an online expressive writing intervention for rural breast cancer survivors: A randomized controlled trial. Eur J Oncol Nurs Off J Eur Oncol Nurs Soc. 2025;74:102790. doi:10.1016/j.ejon.2025.102790 39813977

[15] Clarke V, Braun V. Thematic analysis. J Posit Psychol. 2017;12(3):297–8. doi:10.1080/17439760.2016.1262613

[16] Tedeschi Richard G., Calhoun Lawrence G.. Posttraumatic Growth and Expert Companionship in Grief Therapy ∣ 7 ∣ N. In: New Techniques of Grief Therapy [Internet]. Routledge; 2021 [cited 2025 Apr 15]. Available from: https://www.taylorfrancis.com/chapters/edit/10.4324/9781351069120-7/posttraumatic-growth-expert-companionship-grief-therapy-richard-tedeschi-lawrence-calhoun

[17] Michalczyk J, Dmochowska J, Aftyka A, Milanowska J. Post-Traumatic Growth in Women with Breast Cancer: Intensity and Predictors. Int J Environ Res Public Health. 2022;19(11):6509. doi:10.3390/ijerph19116509 35682111 PMC9180473

[18] Borders TF. Satisfaction With Care Among Cancer Survivors With Medicare Coverage: Are There Rural Versus Urban Inequities? J Prim Care Community Health. 2024;15:21501319241240342. doi:10.1177/21501319241240342 38523417 PMC10962042

[19] Smith-Turchyn J, Allen L, Dart J, Lavigne D, Rooprai S, Dempster H, Characterizing the Exercise Behaviour, Preferences, Barriers, and Facilitators of Cancer Survivors in a Rural Canadian Community: A Cross-Sectional Survey. Curr Oncol Tor Ont. 2021;28(4):3172–87. doi:10.3390/curroncol28040276 PMC8395505 34436042

[20] DeGuzman PB, Vogel DL, Bernacchi V, Scudder MA, Jameson MJ. Self-reliance, Social Norms, and Self-stigma as Barriers to Psychosocial Help-Seeking Among Rural Cancer Survivors With Cancer-Related Distress: Qualitative Interview Study. JMIR Form Res. 2022;6(5):e33262. doi:10.2196/33262 35588367 PMC9164097

[21] Kuzmickus D, Kang Balzarini T. Close to home: Intersectionality of familiarity, emotions, and stigma of mental disorders among rural residents. SSM - Qual Res Health. 2025;7:100572. doi:10.1016/j.ssmqr.2025.100572

[22] Morris BB, Rossi B, Fuemmeler B. The role of digital health technology in rural cancer care delivery: A systematic review. J Rural Health Off J Am Rural Health Assoc Natl Rural Health Care Assoc. 2022;38(3):493–511. doi:10.1111/jrh.12619 PMC8894502 34480506

[23] Watson J, Peng CS, Desir M, Mate-Cole MN, Amonoo H. Expressive writing interventions in patients with cancer: A scoping literature review. Palliat Support Care. 2025;23:e132. doi:10.1017/S1478951525100394 40686255 PMC13166703

[24] Pennebaker JW. The Social, Linguistic, and Health Consequences of Emotional Disclosure. In: Social Psychological Foundations of Health and Illness [Internet]. 2003 [cited 2024 Apr 17]. p. 288–313. Located at: pub.1037638462. Available from: https://app.dimensions.ai/details/publication/pub.1037638462 doi:10.1002/9780470753552.ch11

[25] Lai J, Song H, Wang Y, Ren Y, Li S, Xiao F, Efficacy of expressive writing versus positive writing in different populations: Systematic review and meta-analysis. Nurs Open. 2023;10(9):5961–74. doi:10.1002/nop2.1897 37434395 PMC10415981

[26] Sargunaraj M, Kashyap H, Chandra PS. Writing Your Way Through Feelings: Therapeutic Writing for Emotion Regulation. J Psychosoc Rehabil Ment Health. 2021;8(1):73–9. doi:10.1007/s40737-020-00198-1

[27] Menger F, Mohammed Halim NA, Rimmer B, Sharp L. Post-traumatic growth after cancer: a scoping review of qualitative research. Support Care Cancer Off J Multinatl Assoc Support Care Cancer. 2021;29(11):7013–27. doi:10.1007/s00520-021-06253-2 PMC8464569 34018030

[28] Pennebaker JW, Seagal JD. Forming a story: the health benefits of narrative. J Clin Psychol. 1999;55(10):1243–54. doi: 10.1002/(SICI)1097-4679(199910)55:10<1243::AID-JCLP6>3.0.CO;2-N 11045774

[29] Smith J. Psychotherapy: A Practical Guide [Internet]. 2017 [cited 2025 Apr 15]. Available from: https://link.springer.com/book/10.1007/978-3-319-49460-9

[30] Almeida M, Ramos C, Maciel L, Basto-Pereira M, Leal I. Meaning in life, meaning-making and posttraumatic growth in cancer patients: Systematic review and meta-analysis. Front Psychol. 2022;13:995981. doi:10.3389/fpsyg.2022.995981 36570997 PMC9784472

[31] Henson C, Truchot D, Canevello A. What promotes post traumatic growth? A systematic review. Eur J Trauma Dissociation. 2021;5(4):100195. doi:10.1016/j.ejtd.2020.100195

[32] Jallo N, Kinser PA, Eglovitch M, Worcman N, Webster P, Alvanzo A, Giving Voice to Women with Substance Use Disorder: Findings from Expressive Writing About Trauma. Womens Health Rep. 2024;5(1):223–30. doi:10.1089/whr.2023.0173 PMC10956529 38516652

[33] Bainbridge C, Bryant G, Dale R. Reconsidering intrapersonal communication through an interdisciplinary lens. Front Psychol. 2025;16:1569493. doi:10.3389/fpsyg.2025.1569493 40861340 PMC12376441

[34] Liu X, Zhang Q, Yu M, Xu W. Patterns of posttraumatic stress disorder and posttraumatic growth among breast cancer patients in China: A latent profile analysis. Psychooncology. 2020;29(4):743–50. doi:10.1002/pon.5332 31957127

[35] Huang S, Huang M, Long F, Wang F. Post-traumatic growth experience of breast cancer patients: A qualitative systematic review and meta-synthesis. PLOS ONE. 2025;20(1):e0316108. doi:10.1371/journal.pone.0316108 39847563 PMC11756777

[36] Blickle P, Schmidt ME, Steindorf K. Post-traumatic growth in cancer survivors: What is its extent and what are important determinants? Int J Clin Health Psychol. 2024;24(1):100418. doi:10.1016/j.ijchp.2023.100418 37867603 PMC10585376

[37] Cregg DR, Cheavens JS. Gratitude Interventions: Effective Self-help? A Meta-analysis of the Impact on Symptoms of Depression and Anxiety. J Happiness Stud. 2021;22(1):413–45. doi:10.1007/s10902-020-00236-6

[38] Zachariae R, O’Toole MS. The effect of expressive writing intervention on psychological and physical health outcomes in cancer patients–a systematic review and meta-analysis. Psychooncology. 2015;24(11):1349–59. doi:10.1002/pon.3802 25871981 PMC6680178

[39] Guo L. Find a Resting Place for Your Emotions and Make it Yours: A Meta-Analysis of Expressive Writing Interventions Among Asian Populations. Cogn Ther Res. 2023;47(6):936–57. doi:10.1007/s10608-023-10417-1

[40] Zhang C, Xu S, Wen X, Liu M. The effect of expressive writing on Chinese cancer patients: A systematic review and meta-analysis of randomized control trails. Clin Psychol Psychother. 2023;30(6):1357–68. doi:10.1002/cpp.2878 37345260

